# Comparison between transrectal palpation, B-mode and Doppler ultrasonography to assess luteal function in Holstein cattle

**DOI:** 10.3389/fvets.2023.1162589

**Published:** 2023-05-04

**Authors:** Uxía Yáñez, Antonio V. Murillo, Juan J. Becerra, Pedro G. Herradón, Ana I. Peña, Luis A. Quintela

**Affiliations:** ^1^Unit of Reproduction and Obstetrics, Department of Animal Pathology, Faculty of Veterinary Medicine, Universidade de Santiago de Compostela, Lugo, Spain; ^2^Friends of Animals Veterinary Hospital Srl, Latina, Italy

**Keywords:** ultrasound, blood flow, rectal palpation, reproduction, corpus luteum, cow

## Abstract

**Introduction:**

Over the years, the most common methods for monitoring reproductive health in cattle have varied from transrectal palpation to B-mode ultrasonography. Nowadays, some portable ultrasound equipment includes the Doppler mode. Therefore, the aim of this study was to compare the accuracy of the different methods to assess corpus luteum (CL) functionality.

**Methods:**

In Experiment 1, 53 Holstein lactating cows undergoing a synchronization protocol were examined via transrectal palpation and B-mode scanning. Measurements for the largest diameter (LAD) and subjective size of CL (SCLS) were collected. Data were analyzed using correlation analysis and ROC Curves. In Experiment 2, 30 Holstein non-lactating cows with a CL were administered PGF2α and examined several times after injection, first in B-mode and then with Power Doppler. Measurements for LAD, CL area (CLA) and subjective and objective CL blood flow were collected. Blood samples were taken in both experiments to determine P4 concentration. Data were analyzed using correlation analysis and the GLM repeated measures test.

**Results:**

Results for Experiment 1 showed that LAD was more accurate than SCLS. In Experiment 2, CLA was the best measurement to assess CL function, although both subjective and objective CL blood flow offer accurate information 24 h after PGF2α administration.

**Discussion:**

Consequently, ultrasonography provides more accurate information about CL function than transrectal palpation. Although CLA seems to be an earlier indicator of luteal function than blood flow, 24 h after the onset of luteolysis, both parameters are valid.

## 1. Introduction

Until the end of the 20th century, the most commonly used method by veterinarians to carry out reproductive monitoring in cattle was transrectal examination. Few veterinarians used ultrasonography at that time, although its usefulness was not unknown, as this technique was frequently used in other species (1). In addition, there were also some studies on its use in cattle (2–5). However, the limited availability of portable equipment, as well as their high price, hindered the use of ultrasonography on farms.

It was at the beginning of the 21st century that the interest in ultrasound began to increase, favored by the commercialization of portable equipment at a reasonable price and the numerous training courses on the subject. However, it was not until well into the 21st century that reproductive veterinarians began to use ultrasound routinely. At this time, B-mode was most frequently used, although there were some devices that included the Doppler mode.

Nowadays, there is a wide variety of portable ultrasound equipment, many of which include the Doppler mode, available to veterinarians at affordable prices. Therefore, the question arises as to whether, once the use of B-mode ultrasound has been established, it is worth investing in Doppler equipment and, if so, what advantages it has over B-mode. In order to resolve this, it is necessary to understand the applications for which the B-mode has been used and consider its limitations. The first use of ultrasound equipment in cattle, and the reason why many professionals started to use it, was the pregnancy diagnosis, since it provided a proper level of accuracy as soon as 28 d after insemination through visualization of the embryo. This was great progress compared to the diagnosis by palpation, which used to be performed around 45–60 days post-insemination and was based on the detection of changes in the shape and texture of the uterine horn and the slip of the embryonic sac membranes (6). However, the applications of ultrasound in bovine reproduction are not limited to a simple pregnancy diagnosis.

Ultrasonography is also used to identify the structures present in the ovary, thus facilitating the timing of the cycle, which allows veterinarians to determine ovarian activity in order to decide the most appropriate protocols and even to select embryo recipient animals (4, 7). At a uterine level, it allows for the diagnosis of uterine pathologies that are difficult to detect using transrectal examination, such as endometritis (8, 9). In addition, another great advantage of ultrasound is that it can detect embryonic mortality, fetal malformations and it makes it possible to discover the fetal sex at 55 days of gestation (9, 10).

In addition to all these applications within ovarian exploration, ultrasonography can be used to determine the presence of a corpus luteum (CL). However, one of the limitations of B-mode is that it does not provide information about the activity of the CL. Although palpation makes it possible to identify certain CL characteristics that may offer information about the moment in the cycle, such as size and consistency, a great deal of experience is required to obtain accurate results. Since the main function of the CL is the production of progesterone, the identification of a non-active CL at around 20–21 days post-insemination has been studied as a possible method for early nonpregnancy diagnosis (11, 12). In addition, determining its activity is also useful in order to ascertain the timing of the cow’s cycle and to select the most appropriate synchronization protocols, since a young CL will not respond to prostaglandin F2α (PGF2α), and a regressing CL is already under the influence of endogenous PGF2α (13).

To overcome this limitation, numerous researchers have studied the correspondence between different measurements of the CL and its activity to determine the best method for assessing the functionality of the CL on farms, without the need to determine the progesterone concentration. To this end, the correspondence between progesterone concentration and several CL characteristics in *Bos taurus* (14–16) and other species (17–19) has been studied. Moreover, since it is known that the progesterone production capacity of the CL is related to its vascularization (14, 15, 20, 21), numerous studies have been carried out to test the efficiency of the Doppler mode in performing early nonpregnancy diagnosis as well as the selection of animals for synchronization protocols and the results obtained are quite promising (11, 12, 15, 22).

For this reason, this study aims to compare the different methods used to detect an active CL by veterinarians over the last three decades and give an overview of the changes and progress made during this period. Two experiments were performed: the objective of Experiment 1 was to compare the accuracy of transrectal palpation and B-mode ultrasound; the objective of Experiment 2 was to compare the accuracy of B-mode ultrasound and Doppler ultrasound. Altogether, the aim was to determine the degree of diagnostic accuracy depending on the method used and to assess the need to invest in new equipment.

## 2. Materials and methods

### 2.1. Animals

In Experiment 1, 53 Holstein, nonpregnant, lactating cows (parity 1–3) were enrolled. Cows belonged to one herd located in A Pastoriza (Lugo). It was a mixed tie-stall and grazing farm and housed a total of 80 lactating cows. Mean milk production was 25–35 L/day. In Experiment 2, 30 Holstein cows were included in the study. These were cows belonging to the teaching herd and located in a tie-stall facility at the Rof Codina Veterinary Teaching Hospital (Faculty of Veterinary Medicine, at the Universidade de Santiago de Compostela, Lugo Campus, Spain). As the main purpose of these animals was clinical, none of them were pregnant nor in lactation. All cows were clinically and gynecologically healthy. Both experiments were conducted in accordance with the European and Spanish Regulations for the protection of animals used for scientific purposes (Directive 2010/63/EU, RD 53/2013).

### 2.2. Study design

The selection criteria in Experiment 1 were established according to the reproductive status of the animals. Routine monthly reproductive examinations were carried out by a veterinarian and data was recorded on farm software. Only cycling cows, with 40–85 days in milk which were enrolled in a synchronization protocol, were included. The protocols selected to include cows in this experiment were Ovsynch (*n* = 32) and heat synchronization with prostaglandin F2α (PGF2α, *n* = 21). Those cows under the Ovsynch protocol were explored on the first GnRH day, PGF2α day, second GnRH day, and 19–22 days after artificial insemination (AI). Those cows undergoing heat synchronization with PGF2α were examined on PGF2α day, 5 days later (to check the effect of PGF2α) and 19–22 days after AI. The examination days were selected so that the CL status could be assessed at each hormone administration and at the end of the cycle, i.e., luteal regression, if the cow was not pregnant. For the examination, firstly transrectal palpation was performed, followed by an ultrasonography exam and blood collection. All the examinations were performed by the same experienced veterinarian.

**Figure 1:**
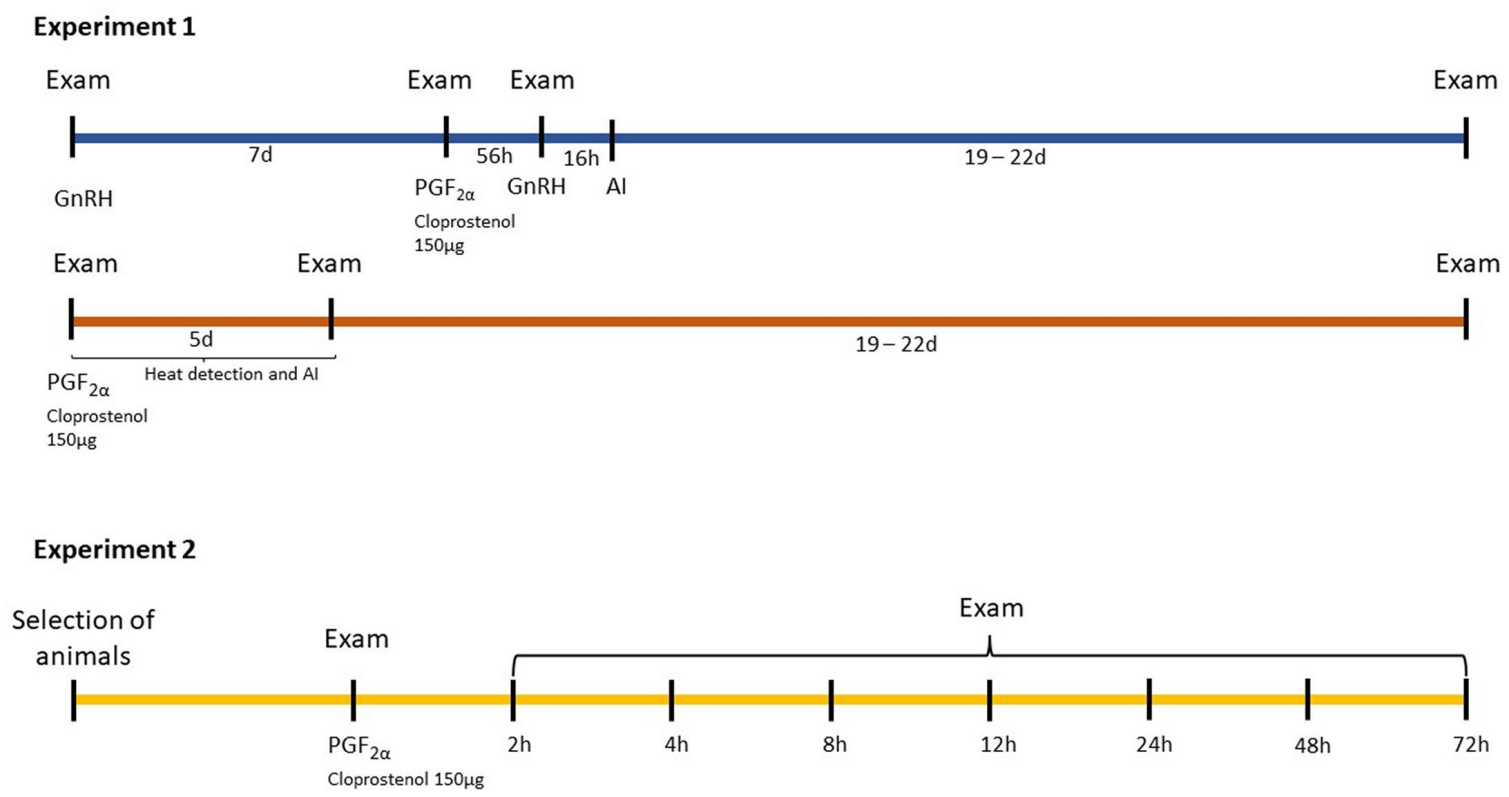
Design of the protocols followed in Experiment 1 and 2 to determine the accuracy of transrectal palpation (P), B-mode (US) and Doppler ultrasonography (DU) to assess luteal function in Holstein cows. Examinations (Exam) consisted of P, US, and blood collection (BC) in Experiment 1, and US, BC, and DU in Experiment 2.

To select the animals in Experiment 2, an ultrasonography exam of the genital tract was performed. The examinations were carried out by two experienced veterinarians. The presence of a CL with a diameter ≥1.8 cm, according to the mean value between those proposed by Donate et al. (23) and Bicalho et al. (24), and a subjective luteal blood flow (SLBF) ≥2 (see Subjective corpus luteum blood flow evaluation scale below) were established as inclusion criteria. The selected cows were treated with intramuscular 150 μg of PGF2α analogue (Cloprostenol, Dalmazin, Fatro Ibérica, Barcelona, Spain). The moment of PGF2α injection was considered the beginning of the experiment (0 h). The time of examinations is depicted in Figure 1.

### 2.3. Ultrasonography and image analysis

Ultrasonography examinations of the ovaries were performed using a portable ultrasound device. In Experiment 1, a PieMedical Tringa Scan (Esaote, Genoa, Italy), equipped with a 5.0 MHz linear-array transducer, was used. The scan was set up as follows: Frequency 5.0 MHz, Gain 20% (scale 0–27%), D 11.2, FPS 36. Scans were performed on the days mentioned above. The presence of a CL was assessed and measurements for the largest diameter (LAD) were taken. Additionally, the size of the CL was evaluated subjectively during transrectal palpation.

**Figure 2:**
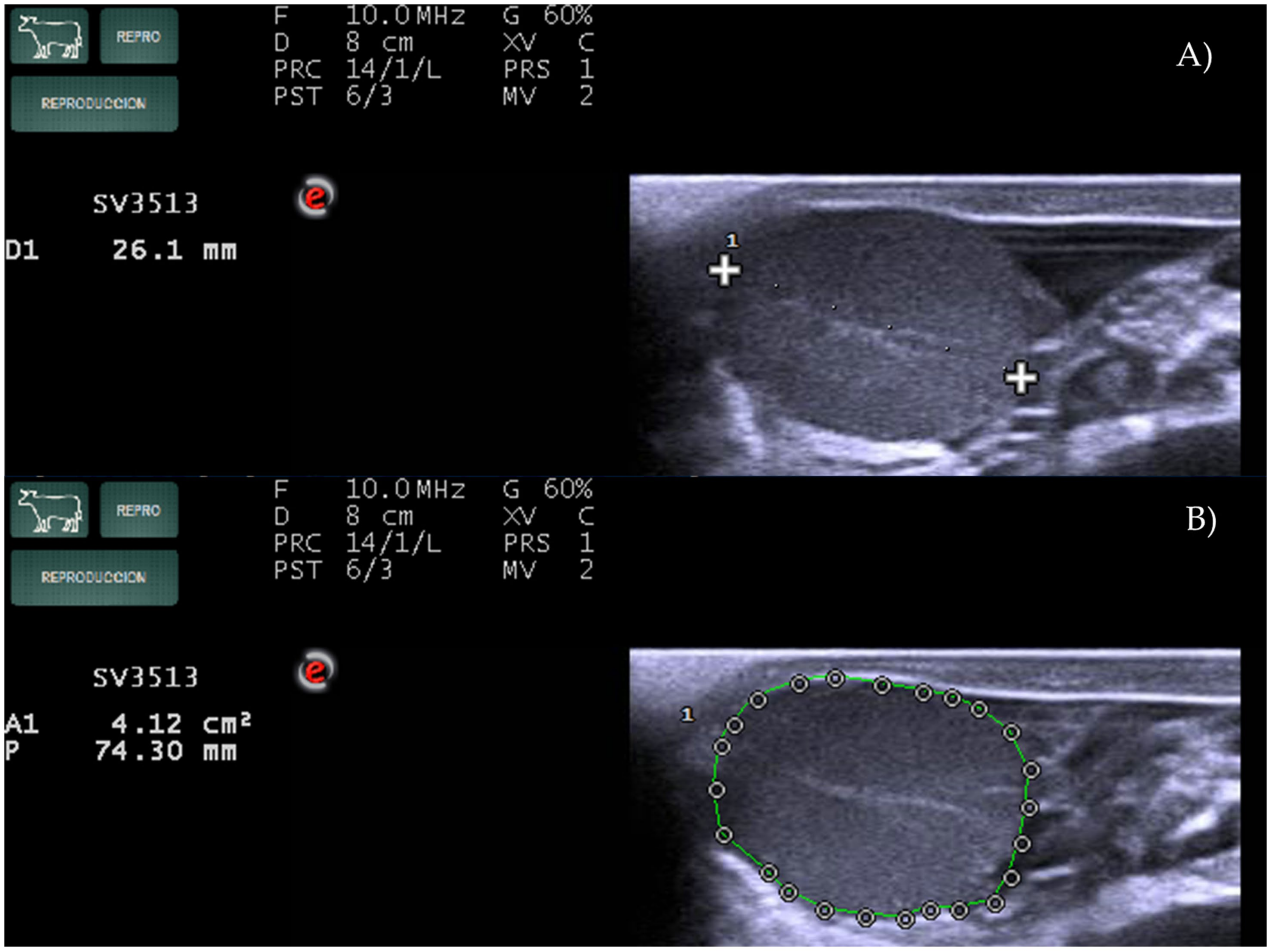
Measurements of **(A)** the largest diameter of the corpus luteum (LAD) and **(B)** corpus luteum area (CLA) using MyLabOne Scan in Holstein cows.

In Experiment 2, a MyLabOne scan (Esaote, Genoa, Italy), equipped with a multifrequency (2–10 MHz), linear-array transducer was used. The scan was set up as follows: Frequency 10.0 MHz, Gain 60%, D 7 cm, PRS 5 for B – mode and Frequency 6.6 MHz, Gain 55%, PRF 500 Hz, PRS 3, PRC M/H for Power Doppler mode. Settings were maintained throughout the experiment. Examinations were performed at 0, 2, 4, 8, 12, 24, 48, and 72 h after the administration of PGF2α. For each animal, two whole scans of the CL were performed, first in B-mode and then with Power Doppler, both recorded individually for their subsequent analysis. All videos were visualized with MyLab Desk software (Esaote, MyLabTM, Genoa, Italy) and processed. On B-mode images, LAD, and CL area (CLA, measured at the largest diameter) were determined (Figure 2).

**Figure 3:**
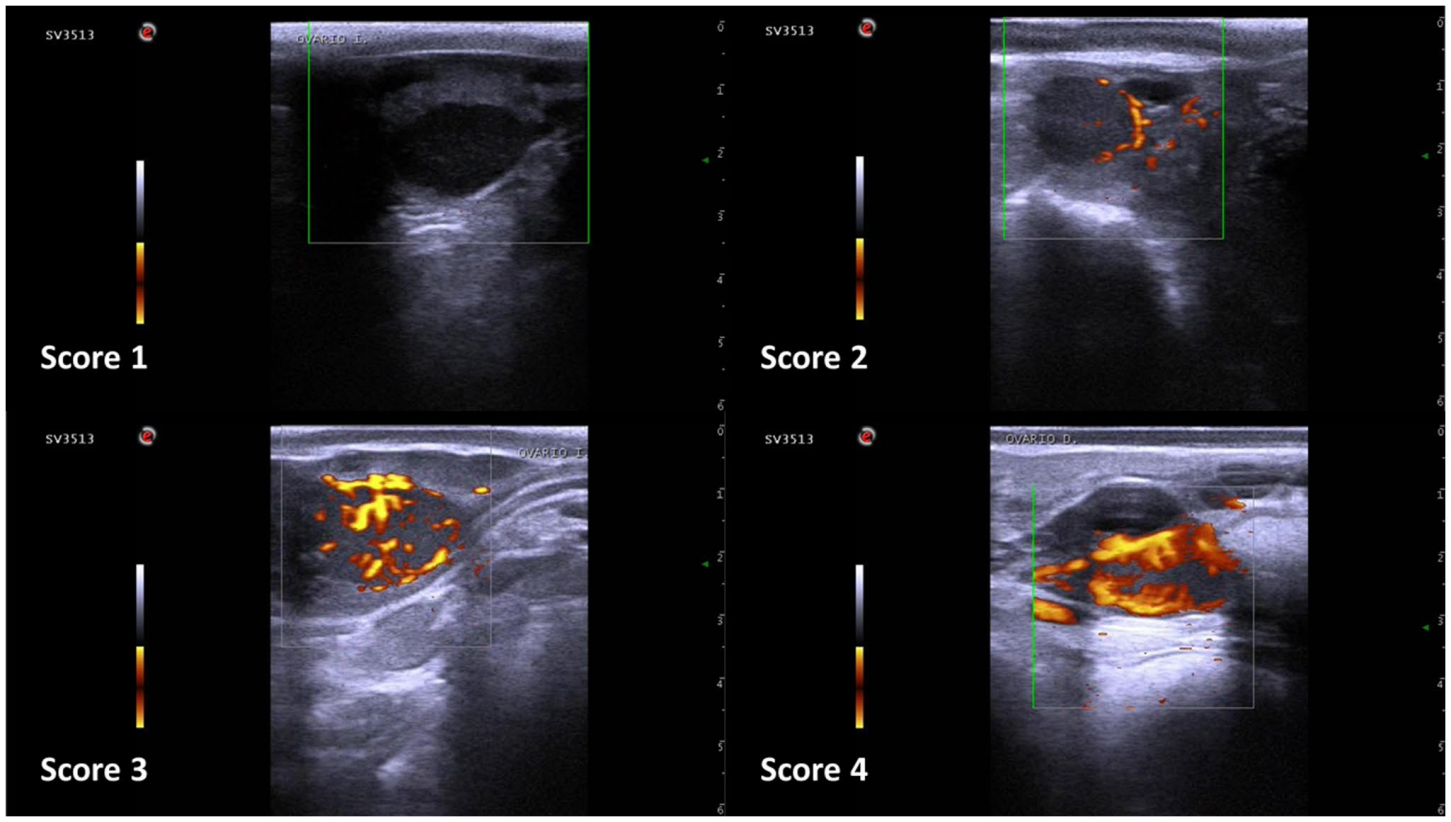
Subjective corpus luteum blood flow evaluation scale in Holstein cows. Score 1: <10% of colored area; score 2: 11–30%; score 3: 31–50%; score 4: >51%.

**Figure 4:**
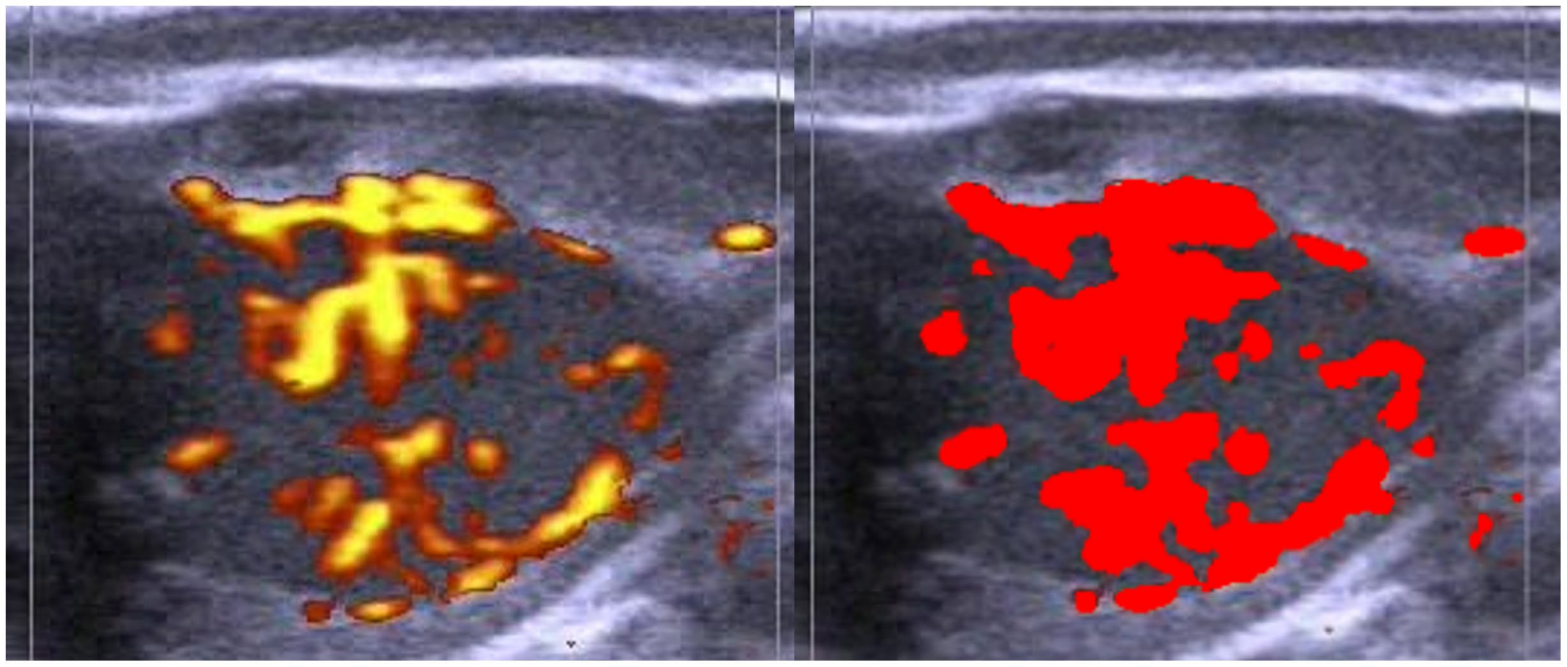
Example of objective corpus luteum blood flow evaluation in Holstein cows using ImageJ software (right). Evaluations were made by recording the area where maximum vascularization was observed (left).

On Power Doppler images, subjective and objective assessments of the CL blood flow (SLBF and OLBF, respectively) were performed. SLBF evaluation was designed according to previous research (12, 25). Estimations were always made by the same person and through visualization of the recording, using a 1–4 scale (Figure 3), where ~<10% of the colored area corresponded to score 1, ~11–30% corresponded to score 2, ~31–50% corresponded to score 3 and ~>51% corresponded to score 4. Conversely, OLBF evaluation was performed by means of ImageJ software (Version 1.46r, Wayme Rasband, National Institutes of Health, USA). Cross sectional images of the CL were extracted from the recordings and the percentage of the colored area was calculated (Figure 4).

### 2.4. Blood samples and progesterone analysis

In both experiments, the determination of progesterone (P4) serum levels were used as the reference method. Blood samples were collected from the coccygeal vein at the end of the examination in Experiment 1, just before the PGF2α administration and immediately prior to each ultrasonography examination in Experiment 2. Samples were kept in refrigeration for 8 h and then centrifugated at 1500 g for 15 min. Serum was separated into 0.5 ml aliquots and frozen at −20°C until P4 was determined.

Serum P4 concentrations were measured with a commercial progesterone ELISA kit (DRG 17-α-OH Progesterone ELISA, DRG Instruments GmbH, Germany), following the manufacturer’s instructions. Optical densities were measured in a microplate reader (Bio-Rad 550 model, Bio-Rad laboratories S.A., Madrid, Spain). The detection limit for P4 ranged from 0.034 to 20 ng/ml, with an analytical sensitivity of 0.034 ng/ml.

### 2.5. Statistical analysis

In Experiment 1, serum P4 concentration and subjective CL size (SCLS) were considered categorical variables. Serum P4 concentration was divided into non-functional CL (≤2 ng/ml), not able to respond to PGF2α administration; and functional CL (>2 ng/ml), able to respond to PGF2α administration. SCLS was divided into four groups: 0, 1, 2, and 3, with 0 being absence of CL and 1, 2, 3 being presence of CL in order of increasing size. LAD was considered as a continuous variable. Correlation analysis and ROC Curves were performed to determine the efficacy of transrectal palpation and ultrasonography exams to identify PGF2α-sensitive CL. Additionally, a cut-off point was established for each technique, and sensitivity (SE), specificity (ES), positive predictive value (PPV), and negative predictive value (NPV) were determined.

In Experiment 2, the categorical variable measured was SLBF, divided into 4 degrees, as previously mentioned. Serum P4 concentration, LAD, CLA and OLBF were considered continuous variables. First, descriptive statistics (mean, standard deviation, 95% confidence interval) and correlations between the different methods were performed. Next, data were analyzed using the General Linear Model (GLM) repeated measures test to determine statistical differences between values obtained at different hours and the one obtained at 0 h. As the units of measurement differed among each of the methods, the percentage of variation with respect to 0 h was calculated according to the following formula:

$$\%\mathrm{variation}=\frac{\mathrm{Value}\;h_{x}-\mathrm{Value}\;h_{0}}{\mathrm{Value}\;h_{0}}\times 100$$

where Value h_x_ is the value obtained at the hour we want to compare and Value h_0_ is the value obtained at 0 h.

In this way, the units of measurement were equalized to allow comparison between the different methods. This comparison was carried out using a GLM repeated measures test and a simple contrasts test to compare the different methods with serum P4 concentration and an analysis of variance (ANOVA) test to compare the percentage of change between methods at each hour. All analyses were conducted in SPSS version 20.0 for Windows (SPSS Inc., Chicago, IL, USA). Differences were considered significant at *p* ≤ 0.05.

## 3. Results

### 3.1. Experiment 1

In Experiment 1, the results for the correlation analysis are summarized in Table 1. For the ROC Curve test, the area under the curve was 0.926 (*p* < 0.01) and 0.815 (p < 0.01) for LAD and SCLS, respectively. The cut-off point determined for LAD was 16 (mm) and the one determined for SCLS was 1. According to these cut-offs, the SE, SP, PPV and NPV were 84.14, 93.67, 93.24, 85.05 and 81.70, 75.94, 77.9 and 80.0 for LAD and SCLS, respectively.

**Table 1:** Experiment 1 results for the correlation of subjective evaluation of corpus luteum size by transrectal palpation (SCLS), largest diameter of CL determined by ultrasonography examination (LAD) and serum P4 concentrations in 53 Holstein cows.

	SCLS	LAD	Serum P_4_
SCLS	-	0.72**	0.47**
LAD		-	0.69**
Serum P_4_			-

** *p* < 0.01.

### 3.2. Experiment 2

In Experiment 2, 27 (90%) of the 30 animals initially enrolled in the study responded to the PGF2α administration and ovulation occurred between 78 and 90 h after PGF2α administration. Results for the correlation analysis are displayed in Table 2. All different measurements are significantly correlated with P4 and among them (*p* < 0.01).

**Table 2:** Experiment 2 results for the correlation analysis between the largest diameter of corpus luteum (LAD), corpus luteum area (CLA), objective luteal blood flow (OLBF), subjective luteal blood flow (SLBF), determined by ultrasonography, and serum P4 concentration (P4), to assess corpus luteum functionality in 30 Holstein cows.

	LAD	CLA	OLBF	SLBF	P4
LAD	-	0.917**	0.501**	0.581**	0.359**
CLA		-	0.564**	0.638**	0.367**
OLBF			-	0.876**	0.296**
SLBF				-	0.301**
P4					-

** *p* < 0.01.

**Figure 5:**
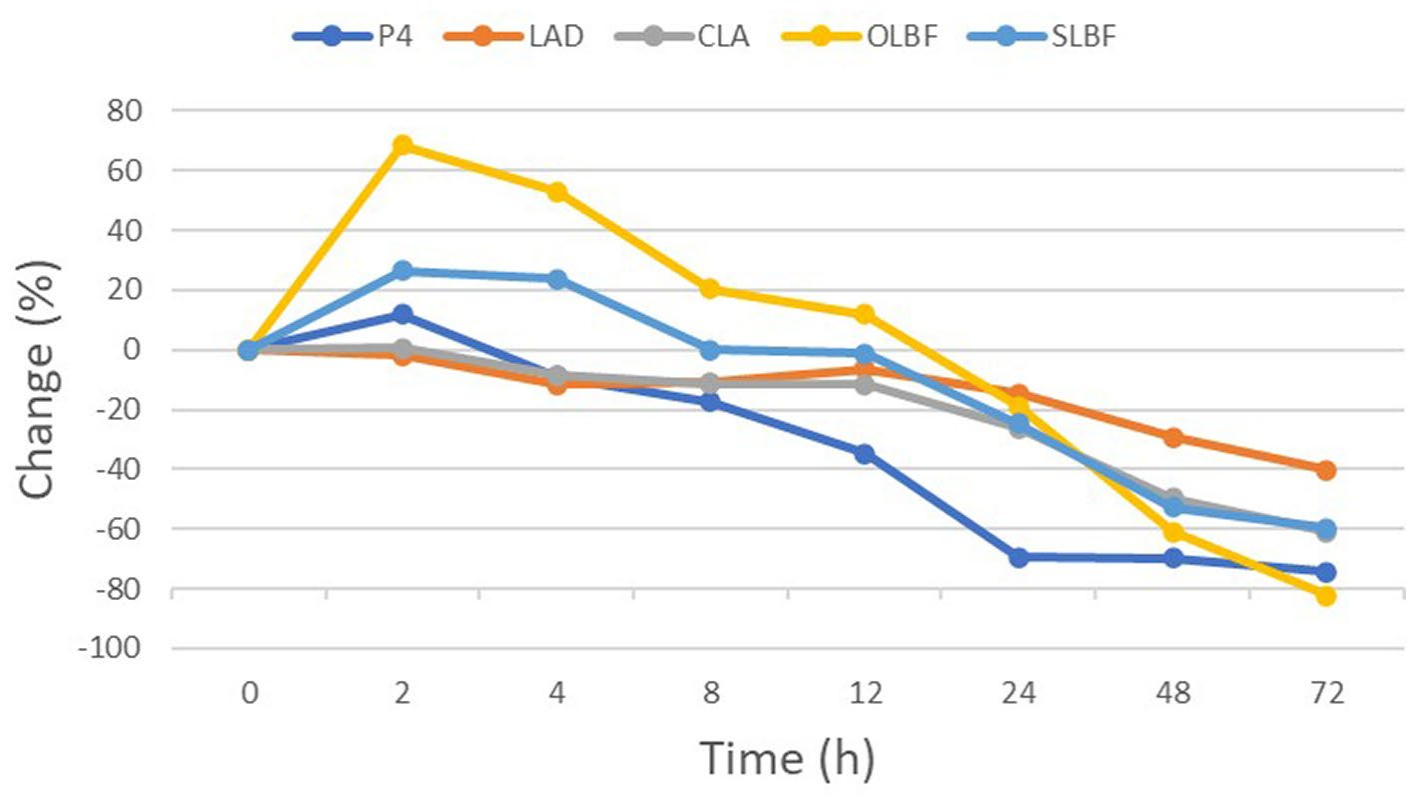
Percentage of change (%), respect to 0 h, of largest diameter of corpus luteum (LAD), corpus luteum area (CLA), objective luteal blood flow (OLBF), subjective luteal blood flow (SLBF) and serum P4 concentration (P4) from 0 to 72 h after PGF2α administration in Holstein cows.

Data for LAD, CLA, OLBF, SLBF, and Serum P4 concentration evolution from 0 to 72 h are displayed in Tables 3 and 4. Percentage of change respect to 0 h is also depicted in Figure 5. LAD declined from 2.58 cm (±0.33) at 0 h to 2.28 (±0.33) cm at 4 h (*p* < 0.05), a reduction of 11.46% (±9.05). CLA decreased from 3.65 (±0.77) at 0 h to 3.31 (±0.87) at 4 h, a diminution of 8.67% (±17.94). OLBF increased from 23.43% (±12.42) at 0 h to 32.72% (±13.13) at 2 h (*p* < 0.05). Thereafter, it diminished and reached a value of 15.61% (±7.00) at 24 h (p < 0.05), which represents a reduction of 18.87% (±7.00) with respect to 0 h. SLBF rose from 2.76 (±0.67) at 0 h to 3.47 (±0.67) at 2 h (p < 0.05); then, it decreased and reached a value of 1.95 (±0.66) at 24 h (p < 0.05), which represents a decline of 24.69% (±0.66). P4 concentration diminished from 8.13 (±5.62) at 0 h to 3.95 (±3.86) at 24 h (p < 0.05), a diminution of 69.45% (±1.59).

**Table 3:** Evolution in Experiment 2 of the largest diameter of corpus luteum (LAD), corpus luteum area (CLA), objective luteal blood flow (OLBF), and subjective luteal blood flow (SLBF), determined by ultrasonography, and serum P_4_ concentration (P4), from 0 h to 72 h after PGF_2α_ administration in 30 Holstein cows.

		LAD (cm)		CLA (cm^2^)		OLBF (%)		SLBF (1–4)		P4 (ng/ml)	
Hour	n	μ	±SD	μ	±SD	μ	±SD	μ	±SD	μ	±SD
0	27	2.58	±0.33	3.65	±0.77	23.43	±12.42	2,76	±0.67	8.13	±5.62
2	27	2.53	±0.37	3.63	±0.78	32.72	±13.13*	3.47	±0.67*	7.29	±8.24
4	27	2.28	±0.33*	3.31	±0.87*	26.10	±12.18	3.33	±0.57*	6.83	±8.36
8	27	2.28	±0.35*	3.16	±0.70*	23.83	±11.11	3.05	±0.74	5.33	±6.39
12	27	2.40	±0.31*	3.15	±0.59*	23.26	±12.87	2.66	±0.96	3.95	±3.86*
24	27	2.19	±0.36*	2.61	±0.81*	15.61	±7.00*	1.95	±0.66*	1.64	±1.59*
48	27	1.81	±0.27*	1.81	±0.53*	7.33	±4.14*	1.24	±0.43*	1.75	±1.69*
72	27	1.53	±0.31*	1.38	±0.53*	2.74	±2.42*	1.04	±0.21*	1.58	±1.91*

* *p* < 0.05 respect to 0 h.

**Table 4:** Percentage of evolution in Experiment 2 of the largest diameter of corpus luteum (LAD), corpus luteum area (CLA), objective luteal blood flow (OLBF), and subjective luteal blood flow (SLBF), determined by ultrasonography, and serum P_4_ concentration (P4), from 0 h to 72 h after PGF_2α_ administration in 30 Holstein cows.

		LAD^B^ (%)		CLA^A^ (%)		OLBF^B^ (%)		SLBF^B^ (%)		P4^A^ (%)	
Hour	n	μ	±SD	μ	±SD	μ	±SD	μ	±SD	μ	±SD
0	27	0.00	±0.00	0.00	±0.00	0.00	±0.00	0.00	±0.00	0.00	±0.00
2	27	-1.96^b^	±7.09	0.48^b^	±14.37	68.48^a^	±95.44	26.54^ab^	±30.75	12.66^ab^	±146.84
4	27	−11.46^b^	±9.05	−8.67^b^	±17.94	52.83^a^	±116.50	23.76^ab^	±36.88	−8.97^b^	±106.63
8	27	−11.08^a^	±0.35	−11.16^a^	±0.70	20.45^a^	±11.11	13.27^a^	±0.74	−17.41^a^	±6.39
12	27	−6.33^ab^	±0.31	−11.49^ab^	±0.59	11.81^a^	±12.87	−1.12^ab^	±0.96	−34.82^b^	±3.86
24	27	−14.65^b^	±0.36	−26.08^b^	±0.81	−18.87^b^	±7.00	−24.69^b^	±0.66	−69.45^a^	±1.59
48	27	−29.14^c^	±0.27	−49.61^b^	±0.53	−60.90^ab^	±4.14	−52.77^ab^	±0.43	−69.74^a^	±1.69
72	27	−40.31^b^	±0.31	−60.81^a^	±0.53	−82.32^ac^	±2.42	−59.82^a^	±0.21	−74.39^ac^	±1.91

^AB^Different letters indicate significant differences between methods *p* < 0.05.

^abc^Different letters indicate significant differences in the same row *p* < 0.05.

## 4. Discussion

Our results for Experiment 1 showed that LAD was more accurate than SCLS to determine CL functionality, which makes ultrasonography a more reliable method than transrectal palpation for that purpose. This is in accordance with previous findings by other researchers (26, 27), who also claimed that ultrasonography has allowed for more accurate diagnosis compared with transrectal palpation in other aspects of reproductive management in cattle, such as pregnancy diagnosis. Transrectal palpation has been the most frequent procedure used for pregnancy diagnosis across the world since the second half of the 20th century. As it does not require expensive equipment or submission of samples to a laboratory, not only is it a cheap technique, but it also enables decisions to be made rapidly. Nevertheless, its limitations should be considered as it is necessary to wait until ~35 days after breeding to perform an accurate pregnancy diagnosis, and it also requires a certain level of experience regarding the estimation of fetus age and the assessment of its viability (28). Moreover, the detection of CL and the determination of its functionality, along with the presence of follicles in the ovary, is also reduced using transrectal palpation and requires a larger size of these structures compared to ultrasonography (27). Nowadays, the potential of ultrasonography over transrectal palpation is widely accepted. The advances achieved with this technology are not limited to pregnancy diagnosis and CL functionality, but also include the collection of follicle data, ultrasonic appearance of the uterus, detection of uterine inflammation, pregnancy characterization, detection of embryonic reabsorption and fetal loss, and detection of twins, among others (1). As a consequence, most veterinarians currently use ultrasonography to perform reproductive management in cattle. Additionally, its use in horses and cattle encouraged the expansion to other species such as sheep, goats, pigs, llamas, and buffalos (16, 29–33).

On the other hand, results for Experiment 2 suggest that, within ultrasonography, CLA would be the best parameter to assess the function of the CL, as this method does not significantly differ from P4 determination, and it shows changes as soon as 4 h after luteolysis induction with PGF2α. Moreover, evaluation of CL blood flow, either objectively or subjectively, can also be used to determine CL function. However, according to our results, the use of this approach should be delayed compared to CLA, as it offers more accurate information 24 h after PGF2α administration. These results are in contrast with the ones reported by Herzog et al. (14), who stated that luteal blood flow is a more appropriate indicator for luteal function. It should be taken into account that a significant peak of blood flow in the CL occurred after the induction of luteolysis in our study, and blood flood did not return to similar levels of those at 0 h until 8 h after PGF2α administration. This phenomenon has also been described by other researchers (21, 34), and it is probably due to the stimulation of nitrogen oxide production in the peripheral arterioles of the mature CL by PGF2α, causing their dilatation and the increase in blood flow.

This peak of vascularization was not confirmed by Herzog et al. (14), and it is likely to be the main reason for the differences between our results and theirs. In fact, we detected this significant increase in luteal blood flow at 2 h after PGF2α with respect to 0 h and, as previously mentioned in this section, it did not return to 0 h values until 8 h. Therefore, a significant diminution of blood flow with regard to those levels at 0 h was not observed until 24 h after induction of luteolysis, which may explain the fact that a significant difference was first obtained using CLA.

Additionally, it has been also stated that B-mode ultrasonography is inaccurate when detecting young and old CL (27). In this regard, the Doppler mode may not offer any advantage for earlier identification of old CL. One possibility might be the use of the acute increase of LBF, one of the earliest physiological signals of the luteolytic cascade (35), as it can be detected using color or power Doppler modes. However, using this increase as an indicator of luteolysis would be rather complicated. It would be necessary to do the examination at the exact moment when the increase occurs. Because it only lasts ~2 h, it would require sequence scans, and a reference of former blood flow would be needed to confirm that there is an increase. Therefore, using Doppler ultrasonography to identify old CL could be an asset only when no or scarce vascularization is present on its surface.

Concerning young CL, it has been claimed that blood flow gradually increases in parallel with the increase in volume and P4 concentration from day 2 to 5, and the extent of angiogenesis reaches a maximum within 2–3 days after ovulation (36–38). Consequently, it is probable that, in this case too, Doppler ultrasonography does not offer any advantage compared to B-mode. In this regard, Rocha et al. (17) reported that CLA correlated most with P4 concentration during CL development, while the correlation of P4 with luteal blood flow was greater during the regression phase.

Regarding luteal blood flow, it is also worth mentioning that similar results were obtained performing objective and subjective evaluations. Moreover, both methods significantly correlated with P4 determination, and the correlation between them was also statistically significant. Additionally, this approach has been previously used by other researchers and has proved to provide excellent accuracy in diagnosing nonpregnancy in dairy cows (11, 12, 39). Furthermore, the fact that a subjective evaluation is simple to perform, causes no harm to the animals, and offers *in situ* information makes this approach a remarkable alternative to the gold standard. Consequently, according to our results, SLBF evaluation could be carried out on the farm to assess CL function and luteal regression as soon as 24 h after the onset of luteolysis.

## 5. Conclusion

In conclusion, ultrasonography provides more accurate information about CL function than transrectal palpation. Within ultrasonography, CLA seems to be a better indicator of luteal function than OLBF and SLBF. Nevertheless, 24 h after the onset of luteolysis, both parameters are valid. In addition, there is a high correlation between OLBF and SLBF, and between both these measurements and P4 determination, which makes SLBF a feasible technique to perform on farms. Therefore, if an ultrasonography scan with the Doppler mode is available, it could be used as a complement to the B-mode examination. However, although the predictions for the future are promising, the applications of Doppler ultrasonography in cattle reproduction need further research. One interesting possibility would be the development of a combined index of CLA and OLBF and its inclusion on ultrasound scans using artificial intelligence, in order to extract information regarding the whole CL scan and determine its functionality in real time and with high accuracy.

## Data availability statement

The raw data supporting the conclusions of this article will be made available by the authors, without undue reservation.

## Ethics statement

The animal study was reviewed and approved by Ethics Committee of the University of Santiago de Compostela. Written informed consent was obtained from the owners for the participation of their animals in this study.

## Author contributions

UY, AM, and LQ participated in the conceptualization, methodology, data curation, and formal analysis. UY and LQ drafted the manuscript. JB, PH, AP, and LQ supervised and revised the manuscript. PH and LQ oversaw project administration and funding acquisition. All authors contributed to the article and approved the submitted version.

## Funding

Uxía Yáñez was funded by Xunta de Galicia (Predoctoral Contract Ref. 2020/122).

## Conflict of interest

Author AM is employed by Friends of Animals Veterinary Hospital Srl. (Italy).

The remaining authors declare that the research was conducted in the absence of any commercial or financial relationships that could be construed as a potential conflict of interest.

## Publisher’s note

All claims expressed in this article are solely those of the authors and do not necessarily represent those of their affiliated organizations, or those of the publisher, the editors and the reviewers. Any product that may be evaluated in this article, or claim that may be made by its manufacturer, is not guaranteed or endorsed by the publisher.
